# Th(IV) Adsorption onto Oxidized Multi-Walled Carbon Nanotubes in the Presence of Hydroxylated Fullerene and Carboxylated Fullerene

**DOI:** 10.3390/ma6094168

**Published:** 2013-09-17

**Authors:** Jing Wang, Peng Liu, Zhan Li, Wei Qi, Yan Lu, Wangsuo Wu

**Affiliations:** 1 Radiochemistry Laboratory, School of Nuclear Science and Technology, Lanzhou University, Lanzhou 730000, China; E-Mails: wangjing2010@lzu.edu.cn (J.W.); liupeng08@lzu.edu.cn (P.L.); qiwei20061987@126.com (W.Q.); 2 Institute of Modern Physics, Chinese Academy of Sciences, Lanzhou 730000, China; E-Mail: lizhaner@126.com; 3 China National Nuclear Corporation Lanzhou Uranium Enrichment Co., Ltd., Lanzhou 730000, China; E-Mail: luyan08@lzu.edu.cn

**Keywords:** influence, C_60_(OH)*_n_*, C_60_(C(COOH)_2_)*_n_*, Th(IV) adsorption, oMWCNTs

## Abstract

The adsorption of Th(IV) onto the surface of oxidized multi-walled carbon nanotubes (oMWCNTs) in the absence and presence of hydroxylated fullerene (C_60_(OH)*_n_*) and carboxylated fullerene (C_60_(C(COOH)_2_)*_n_*) has been investigated. C_60_(OH)*_n_*, C_60_(C(COOH)_2_)*_n_* and oMWCNTs have been chosen as model phases because of their representative in carbon nano-materials family. Adsorption experiments were performed by batch procedure as a function of contact time, pH, ionic strength, and temperature. The results demonstrated that the adsorption of Th(IV) was rapidly reached equilibrium and the kinetic process could be described by a pseudo-second-order rate model very well. Th(IV) adsorption on oMWCNTs was dependent on pH but independent on ionic strength. Adsorption isotherms were correlated better with the Langmuir model than with the Freundlich model. The thermodynamic parameters calculated from temperature-dependent adsorption isotherms suggested that Th(IV) adsorption on oMWCNTs was spontaneous and endothermic. Compared with the adsorption of Th(IV) on the same oMWCNTs free of C_60_(OH)*_n_* or C_60_(C(COOH)_2_)*_n_*, the study of a ternary system showed the inhibition effect of C_60_(OH)*_n_* at high concentration on the adsorption of Th(IV) in a pH range from neutral to slightly alkaline; whereas the promotion effect of C_60_(C(COOH)_2_)*_n_*, even at its low concentration, on Th(IV) adsorption was observed in acid medium.

## 1. Introduction

Multiwalled carbon nanotubes (MWCNTs) have raised much interest during recent years due to their inherent extraordinary structural, mechanical, and electronic properties [1,2,3]. MWCNTs possess some highly desirable sorbent characteristics [4], which make them attractive for a variety of applications including radionuclide adsorption. Previous studies demonstrated that MWCNTs are the promising candidates in the field of chemical aspects of nuclear science and technology for the preconcentration and solidification of lanthanides and actinides from large volume of solutions [5,6,7]. Within the remarkable progress for industrial scale application, the toxicity of MWCNTs must be considered. Once they are released into the environment, the human health and environmental may suffer serious risks. CNTs can damage the plants, animals and human by entering the cells for their nanoscales [8,9,10]. The study showed that the toxicity of MWCNTs not only from itself but also from its adsorption substance. Therefore, understanding the adsorption of radionuclide on MWCNTs is crucial [11].

Thorium finds its extensive application as nuclear fuel in power plants and their main sources are soil, rocks, plants, sand and water. Nuclear spent fuels generally contain actinides like thorium, uranium and various fission products [12]. Th(IV) and its compounds are highly toxic that may cause acute toxicological effects for human leading to potential occupational carcinogens [13] and progressive or irreversible renal injury [14,15]. Thorium exists only stable as Th(IV) in solution, which is an important model element for other tetravalent actinides such as Np(IV), U(IV), and Pu(IV). It is also profit as a tracer for studying environmental important processes [16]. Based upon these importance of Th(IV), the determination of Th(IV) in environmental and biological samples is considerable potential as a tool for assessment of human exposure [15]. The fate and transport of Th(IV) in the environment is generally controlled by sorption reactions, complexation, colloid formation, *etc.* [17]. In recent years, the adsorption of Th(IV) on various materials has been studied extensively [18,19]. However, the adsorption behavior of actinide and lanthanide ions onto oMWCNTs is investigated in a limited number of studies [5,6,7,11,20,21], and the sorption mechanism is still unclear, especially in the presence of other nano-materials.

Fullerenes (C_60_) is another kind of nanometer carbon materials whose discovery is earlier than carbon nanotubes. Due to its definite structure and π electron character, C_60_ is very easy to form π–π stacking interactions with another material equipped with aromatic rings [22,23], which may affect the adsorption of metal ions or organic matter on that material. Therefore, the research about the effect of fullerene on the adsorption of Th(IV) onto oMWCNTs is necessary. However, it is difficult to separate completely two kinds of carbon nano-materials in the identical phase, and to clarify their effect on the adsorptive interaction between carbon nano-materials and Th(IV). Herein, the author choose soluble fullerene to study its influence on the adsorption of Th(IV) onto oMWCNTs. It is essential to research the adsorption of soluble fullerene and Th(IV) on oMWCNTs for understanding the radionuclide pollution and the potential impact evaluation of CNTs on radionuclide behavior.

## 2. Results and Discussion

### 2.1. Characterization of Purified oMWCNTs, C_60_(OH)_n_ and C_60_(C(COOH)_2_)_n_

**Figure 1:**
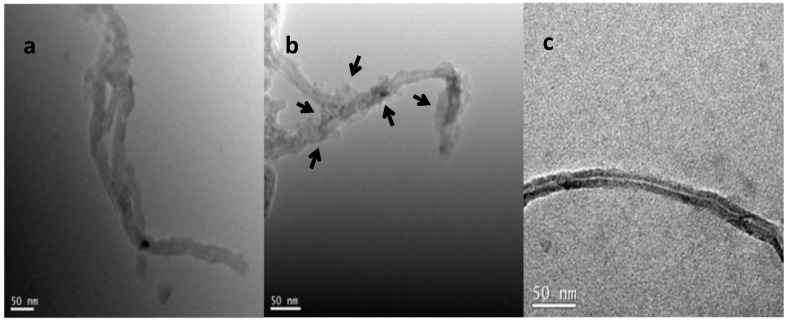
Transmission electron microscope (TEM) images of (**a**) oxidized multi-walled carbon nanotubes (oMWCNTs); (**b**) C_60_(OH)*_n_*-adsorbed oMWCNTs; **(c**) C_60_(C(COOH)_2_)*_n_*-adsorbed oMWCNTs.

The transmission electron microscope (TEM, Hitachi Model H-600, Japan Hitachi company) was used to observe the changes in morphological features of oMWCNTs before and after two kinds of soluble fullerene adsorption. TEM image of oxidized MWCNTs (Figure 1a) display that carbon nanotubes have smooth surface and integrated hollow tubular structure. It can be seen from Figure 1b that C_60_(OH)*_n_* particles (see the narrow present) spread heterogeneously on the surface of oMWCNTs for the C_60_(OH)*_n_*-adsorbed oMWCNTs. Figure 1c indicates that C_60_(C(COOH)_2_)*_n_* particles does not attach with oMWCNTs.

**Figure 2:**
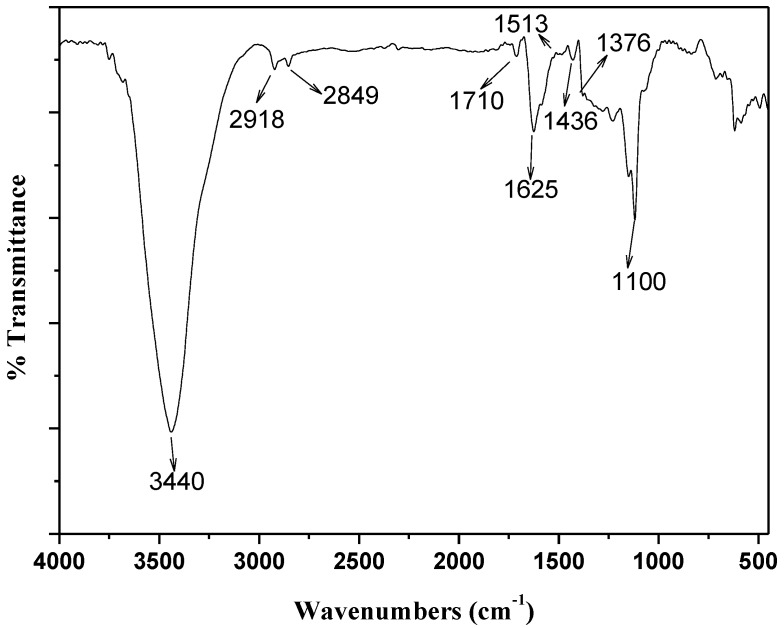
Fourier transform infrared (FTIR) spectra of oMWCNTs.

Fourier transform infrared (FTIR, Nexus670, Thermo Nicolet, American) technique was used in the analysis of the chemical surface groups of the adsorbents. Figure 2 illustrates the FT-IR spectrum of the oxidized carbon nanotubes. The peak at 3440 cm^−1^ is attributed to –OH stretching mode. The peaks at 2918 and 2849 cm^−1^ assigned to asymmetric and symmetric –CH stretching bands. The C=O stretching vibrations peak are appeared at 1710 cm^−1^. The peak at 1625 and 1436 cm^−1^ assigned to C=C stretching bands. The peak at 1513 cm^−1^ can be attributed the carboxylic and carboxylate anion stretching mode. The peak between 1100 and 1376 cm^−1^ is associated with C–O stretching and –OH bending modes of alcoholic, phenolic and carboxylic groups [24,25].

**Figure 3:**
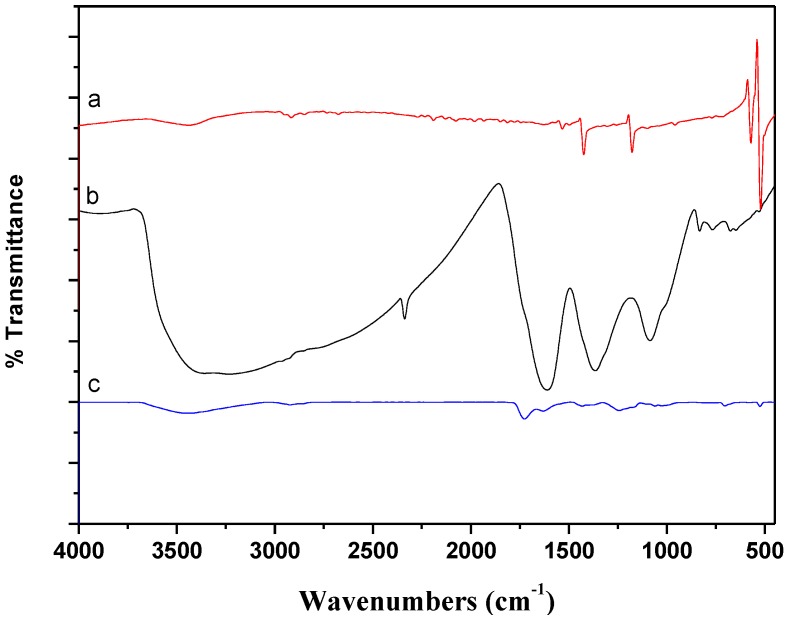
FTIR spectra of the samples: (**a**) raw C_60_; (**b**) C_60_(OH)*_n_*; (**c**) C_60_(C(COOH)_2_)*_n_*.

Figure 3 shows the FT-IR spectrum of raw C_60_, C_60_(OH)*_n_* and C_60_(C(COOH)_2_)*_n_*. For C_60_(OH)*_n_* (Figure 3b), the FTIR spectrum exhibit main peaks at wavenumbers near 3234, 1609, 1365 and 1086 cm^−1^, which are associated with –OH, C=C and –C–O groups [26]. The bands at wavenumbers of 3439, 1718, 1201 and 523 cm^−1^ for C_60_(C(COOH)_2_)*_n_* (Figure 3c) are indicative of the appearance of the stretching of hydroxyl (–OH), carbonyl (>C=O) and carboxyl (–C–O) groups, respectively [27].

### 2.2. Adsorption of Th(IV) onto oMWNTs Surface

#### 2.2.1. The Sorption Kinetics

**Figure 4:**
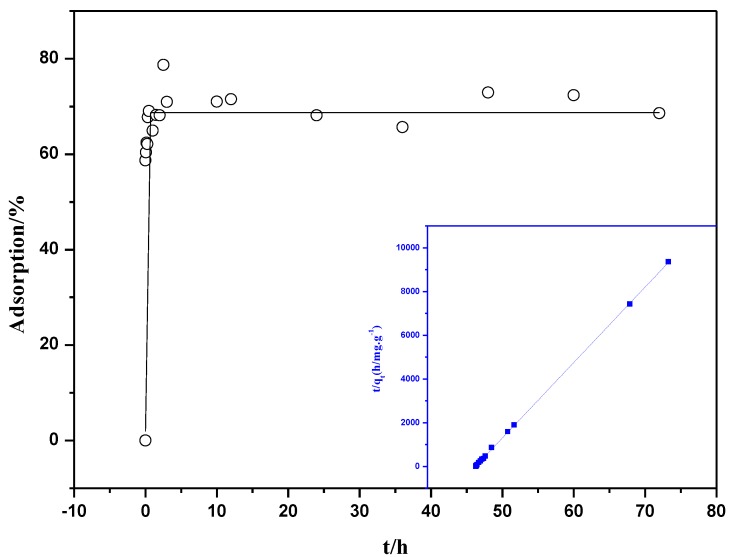
Effect of contact time on Th(IV) adsorption rate onto oMWCNTs and test of pseudo-second-order adsorption kinetics plot for Th(IV), *m/V* = 0.1 g/L, *T =* 25 ± 1 °C, C[Th^4+^] initial = 8.86 × 10^−5^mol/L, *I* = 0.01 mol/L NaNO_3_, pH = 3.20 ± 0.05.

The influence of contact time on the Th(IV) adsorption by oMWNTs was studied. The initial Th(IV) concentrations was 8.86 × 10^−5^ mol/L in the kinetic study. As seen from Figure 4, the uptake of Th(IV) onto oMWCNTs is rapid at the initial contact time, and then the adsorption equilibrium is reached within 3 h. The quick adsorption of Th(IV) suggests that chemical adsorption rather than physical adsorption contributes to Th(IV) adsorption onto oMWCNTs surface [28]. To analyze the adsorption rate of Th(IV) onto oMWCNTs, the pseudo-second-order rate equation (Equation (1)) [29] is employed to simulate the kinetic adsorption data. Its general form is

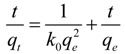
(1)
where *q_t_* (mg/g) is the amount of Th(IV) adsorbed on oMWCNTs at time t and *q_e_* (mg/g) is the equilibrium sorption capacity. *k_o_* (g/mg∙h^−1^) is the pseudo-second-order rate constant of sorption. The straight-line plots of *t/q_t_ vs. t* (Figure 4) is plotted and values of *k_o_* (1.81 × 10^−3^ g/mg∙h^−1^) and *q_e_* (6.44 × 10^−3^ mg/g) are calculated from the intercept and slope of Equation (1). The correlation coefficient of the pseudo-second-order rate equation for the linear plot is 0.9999, which suggests that the kinetic sorption can be described by the pseudo-second-order rate equation very well. Based on the above results, an equilibrium time is selected as 24 h for all subsequent batch experiments.

#### 2.2.2. Influence of pH and Ionic Strength

**Figure 5:**
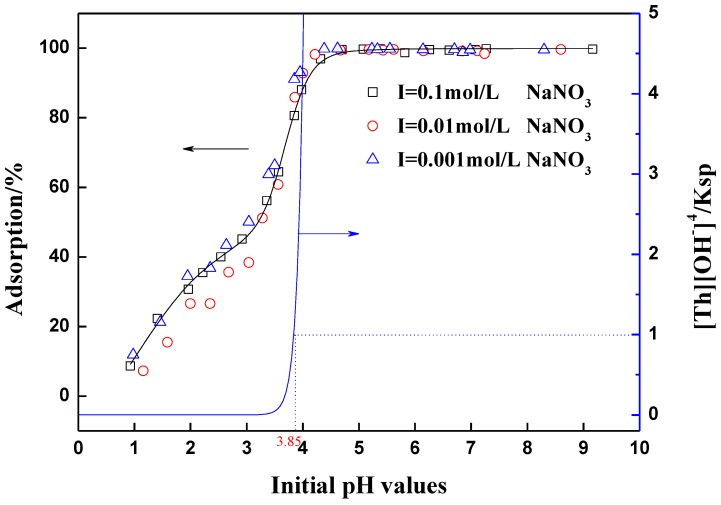
Adsorption of Th(IV) on oMWCNTs as a function of pH, *m/V* = 0.1 g/L, *T =* 25 ± 1 °C, C[Th^4+^] initial = 8.86 × 10^−5^ mol/L.

The pH of solution is a controlling factor in Th(IV) adsorption experiments. Figure 5 shows the effect of pH on Th(IV) removal by varying the pH from1 to 10 in 0.001, 0.01 and 0.1 mol∙L^−1^ NaNO_3_ solution. As shown in Figure 5, Th(IV) adsorption is strongly influenced by the solution pH values. As pH increased from 1 to 4, the fraction of Th(IV) adsorbed onto oMWCNTs also increased, and then the adsorption of Th(IV) maintains at the maximum value of 92% after pH ≥ 4. The maximum adsorption in the pH range 3–4 may be due to the formation of Th(IV) complexes with carboxyl groups on the surface of oMWCNTs. It is well known that oxidation of carbon surface can offer not only more hydrophilic surface structure, but also a large number of oxygen-containing functional groups like –COOH, –OH, or –C=O on the surfaces of oMWCNTs, which increase the sorption capability of carbon material [5]. The results are similar to the Th(IV) adsorption on anatase [30] and silica [31]. The increase of Th(IV) adsorption on oMWCNTs with increasing of pH may be attributed to the surface properties of oMWCNTs in terms of surface charge and dissociation of functional groups [32]. The surface of oMWCNTs contains a large number of binding sites, which may become positively charged at low pH due to the protonation reaction on the surfaces (*i.e.*, $\text{SOH}+{\text{H}}^{+}\Leftrightarrow \text{SO}{\text{H}}_{2}^{+}$). The point of zero charge (pH_pzc_) of oMWCNTs is about 5 [6,33]. At pH < 5, the surfaces were positively charged, and at pH > 5, the surface were negatively charged. The electrostatic repulsion occurred between Th(IV) and the edge groups with positive charge (SOH_2_^+^) on oMWCNTs surface results in the low adsorption efficiency of Th(IV). At high pH values, the surface of oMWCNTs becomes negatively charged due to the deprotonation process (*i.e.*, $\text{SOH}\Leftrightarrow {\text{SO}}^{-}+{\text{H}}^{+}$) and electrostatic repulsion decreases with increasing of pH because of the reduction of positive charge density on the adsorption edges, which enhances the adsorption of the positively charged Th(IV) ions through electrostatic attraction. The characteristic of Th(IV) complex that predominates at a specific solution pH may also play an important role in the uptake efficiency of oMWCNTs for Th(IV) adsorption [34]. According to the hydrolysis constants of Th(IV) (log*k*_1_ = −3.86, log*k*_2_ = −11.82, log*k*_3_ = −24.81, log*k*_4_ = −41.97) [35,36], Th(IV) species present in the forms of Th^4+^, Th(OH)^3+^, Th(OH)_2_^2+^, Th(OH)_3_^3+^, Th(OH)_4_ at different pH values. As can be seen from Figure 5, it is clear that Th(IV) starts to form precipitation at pH = 3.85 if no Th(IV) is adsorbed on oMWCNTs. However, one can see that more than 90% Th(IV) has been adsorbed onto oMWCNTs at pH < 3.85. Therefore, the abrupt increasing of Th(IV) adsorption is not attributed to the precipitation of Th(OH)_4_(s). Sheng *et al.* [37] studied the adsorption of Th(IV) on alumina and they did not think the precipitation is the removal mechanism of alumina toward Th(IV) at pH < 4. They pointed out that the high adsorption of Th(IV) on the surface of oMWCNTs may result in the precipitation of Th(IV) on oMWCNTs due to the local high concentration of Th(IV) on oMWCNTs surface [38,39].

Figure 5 also shows the adsorption of Th(IV) onto oMWCNTs as a function of ionic strength at pH 1–10. Th(IV) adsorption is independent of the ionic strength, which suggests that inner-sphere surface complexes has been formed under this experimental condition [6,11]. Therein, ion exchange does not contribute to the adsorption of Th(IV) onto oMWCNTs. In general, strong dependency on pH and weak dependency on ionic strength, are consistent with inner-sphere complexation [40]. The results of this work are identical to those derived from the literature [12,13,41], although different adsorbate or adsorbents are investigated in the present work.

#### 2.2.3. Influence of the Concentration of oMWCNTs

**Figure 6:**
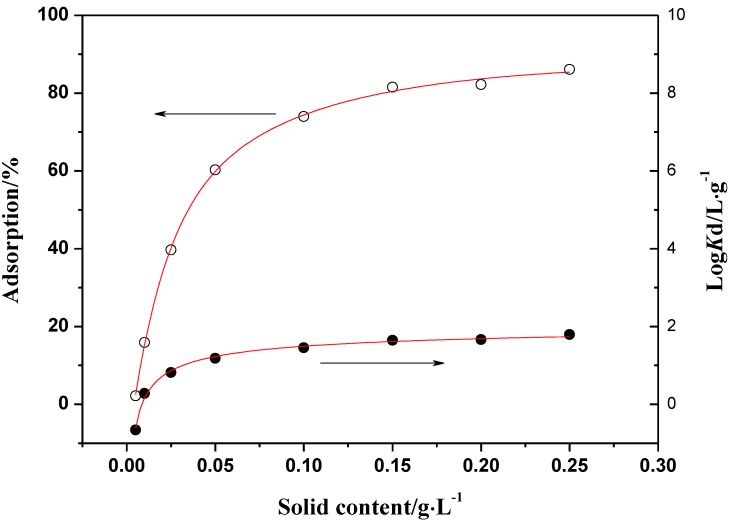
Effect of solid content on the adsorption of Th(IV) onto oMWCNTs, pH = 3.20 ± 0.05, *T =* 25 ± 1 °C, C[Th^4+^] initial = 8.86 × 10^−5^ mol/L, *I* = 0.01 mol/L NaNO_3_.

Adsorption of Th(IV) as a function of oMWCNTs content is shown in Figure 6. The distribution coefficient (*K_d_*) was calculated from the initial concentration of Th(IV) (*C*_0_) and the equilibrium one (*C_e_*) according to the following equation:

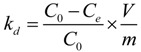
(2)
where *V* is the volume of the solution and *m* is the mass of oMWNCTs. One can see that the sorption percentage of Th(IV) increases with increasing content of oMWCNTs in the system. The functional sites on oMWCNTs surfaces increase with increasing of oMWCNTs content, therefore the sorption percentage of Th(IV) increases reasonably. However, the *K_d_* value increases slightly with increasing content of oMWCNTs. The results of this work are very similar to the literature [42].

#### 2.2.4. Adsorption Isotherms

**Figure 7:**
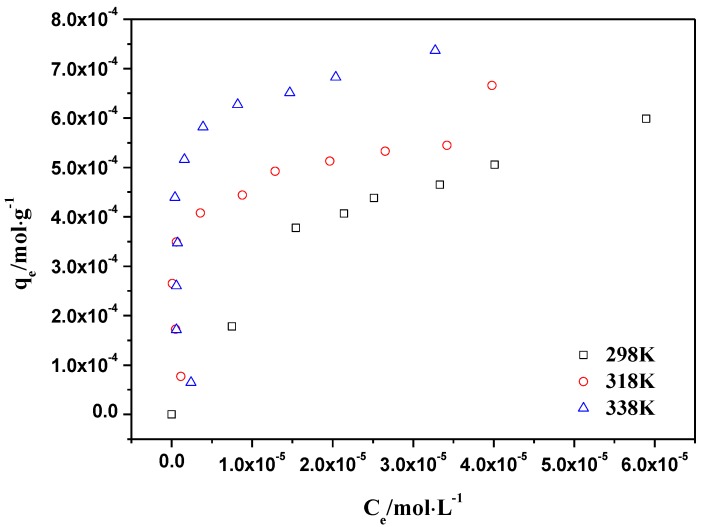
Adsorption isotherms of Th(IV) on oMWCNTs at three different temperatures, *m/V* = 0.1 g/L, pH = 3.20 ± 0.05, *I* = 0.01 mol/L NaNO_3_, C[Th^4+^] initial = 8.86 × 10^−5^ mol/L.

The effect of temperature on the adsorption of Th(IV) was studied by performing a series of experiments at 298, 318 and 338 K. The concentration of Th(IV) varied from 0 to 8.86 × 10^−4^ mol/L. Figure 7 shows the Th(IV) adsorption isotherm on oMWCNTs. The Langmuir model and Freundlich model were used to analyze regressively the adsorption isotherms of Th(IV). The linear form of the Langmuir model can be represented by the following equation:

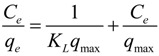
(3)
where *q_e_* (mg/g) and *C_e_* (mg/L) are respectively for the concentration of the metal ions in the solid and liquid phases in equilibrium adsorption, *K_L_* (L/g) is the adsorption equilibrium constant, *q_max_* (mg/g) is the maximum adsorption capacity of the metal ion.

The Freundlich isotherm has the following linear form:

log *q_e_* = log *K_F_* + *n*log *C_e_* (4)

where *q_e_* (mg/g) represents the adsorption amount of the metal ions in the unit mass of oMWCNTs; *C_e_* (mg/L) is the equilibrium aqueous concentration; *K_F_* and *n* are the absorption capacity and strength in the Freundlich the equilibrium constants, respectively.

**Figure 8:**
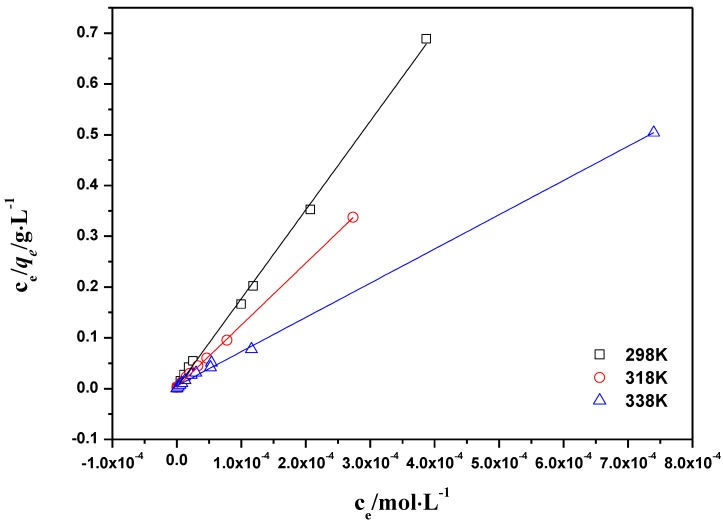
Langmuir model of Th(IV) adsorption onto oMWCNTs, *m/V* = 0.1 g/L, pH = 3.20 ± 0.05, *I* = 0.01 mol/L NaNO_3_, C[Th^4+^] initial = 8.86 × 10^−5^ mol/L.

**Figure 9:**
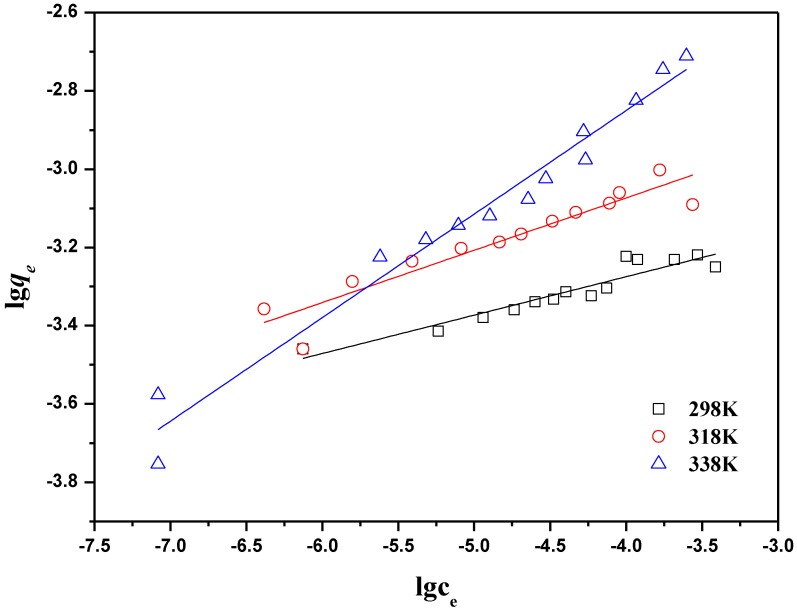
Freundlich model of Th(IV) adsorption onto oMWCNTs, *m/V* =0.1 g/L, pH = 3.20 ± 0.05, *I* = 0.01 mol/L NaNO_3_, C[Th^4+^] initial = 8.86 × 10^−5^ mol/L.

The linearized Langmuir and Freundlich plots are shown in Figure 8 and Figure 9. Parameter values for two kinds of models are presented in Table 1. The Langmuir model showed a higher correlation coefficient than the Freundlich model. This is consistent with the results in the literature, which shows that the adsorption of Th(IV) on the other materials meet the Langmuir model better [43]. The thermodynamic parameters of ∆*H*^0^, ∆*G*^0^ and ∆*S*^0^ calculated from different adsorption isotherms are tabulated in Table 2. The ∆*H*^0^ value was positive as expected for an endothermic reaction under the conditions applied. Figure 7 also confirmed that the adsorption of Th(IV) on oMWCNTs increases with increasing temperature. The negative ∆*G*^0^ value indicated that the adsorption process is spontaneous for Th(IV) adsorption. The decrease in ∆*G*^0^ with the increase of temperature exhibited more efficient adsorption at higher temperature [5].

**Table 1:** The parameters of Langmuir and Freundlich fitting of Th(IV) adsorption on oMWCNTs.

T(K)	Langmuir constants			Freundlich constants		
	*q_max_* (mg·g^−1^)	*K_L_*	*R^2^*	*K_F_* (mg^1−*n*^·L^n^·g^−1^)	*n*	R^2^
298	5.72× 10^−4^	423.7	0.998	1.31× 10^−3^	0.09	0.887
318	8.18× 10^−4^	359.7	0.999	2.89× 10^−3^	0.13	0.881
338	1.48× 10^−3^	185.2	0.998	1.62× 10^−3^	0.26	0.969

**Table 2:** Values of thermodynamic parameters for the adsorption of Th(IV) on oMWCNTs.

*C*_0_ (mg·L^−1^)	Δ*H*^0^ (KJ·mol^−1^)	Δ*S*^0^ (J·mol^−1^·K^−1^)	Δ*G*^0^ (KJ·mol^−1^)		
			298 K	318 K	338 K
7.09 × 10^−5^	42.65	168.12	−7.19	−11.47	−13.84
7.98 × 10^−5^	39.52	154.88	−6.53	−10.03	−12.69
8.87 × 10^−5^	33.67	134.09	−6.17	−9.28	−11.51
1.06 × 10^−4^	32.15	125.75	−5.17	−8.23	−10.15

### 2.3. Influence of C_60_(OH)_n_ on the Adsorption of Th(IV)

#### 2.3.1. Adsorption of Th(IV) *vs.* pH in the Presence of C_60_(OH)*_n_*

**Figure 10:**
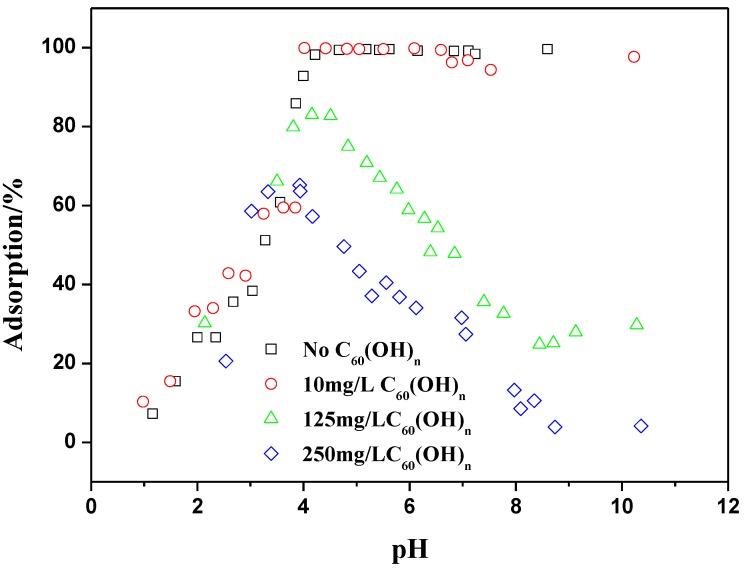
Effect of C_60_(OH)*_n_* on Th(IV) adsorption onto oMWCNTs as a function of pH, *m/V* = 0.1 g/L, *T =* 25 ± 1 °C, *I* = 0.01 mol/L NaNO_3_, C[Th^4+^] initial = 8.86 × 10^−5^ mol/L.

Figure 10 presents Th(IV) adsorption onto oMWCNTs in the absence and presence of C_60_(OH)*_n_* as a function of pH values. The concentration of C_60_(OH)*_n_* is fixed from 10 to 250 mg/L. The adsorption curve of Th(IV) in ternary system presented in Figure 10 can be divided into two different parts. Within the first region (1 ≤ pH ≤ 4), the adsorption of Th(IV) increases with increasing pH in the presence of C_60_(OH)*_n_*. However, the adsorption hindrance of Th(IV) appears in the presence of high concentration of C_60_(OH)*_n_* (*i.e.*, 125 and 250 mg/L) at pH 4. The second region lies where the adsorption of Th(IV) markedly decreases with increasing pH when pH exceeds 4 in ternary system. A striking observation can be made that the decreases of Th(IV) adsorption is correlated strongly to the concentration of C_60_(OH)*_n_*. The adsorption curve of Th(IV) in the presence of 10 mg/L C_60_(OH)*_n_* is similar to that in the absence of C_60_(OH)*_n_*. When the concentration of C_60_(OH)*_n_* is 125 mg/L, the adsorption of Th(IV) decreases from approximate 83% at pH 4 to 20% at pH 8.5. While the adsorption of Th(IV) decreases from nearly 63% at pH 4 to 3% at pH 8.5 when 250 mg/L C_60_(OH)*_n_* is added. It can be stated that C_60_(OH)*_n_* impedes obviously the adsorption of Th(IV) onto oMWCNTs. These observations are not in agreement with the general behavior of Th(IV) in natural systems, which are associated with organic matter [17,44,45,46].

**Figure A1:**
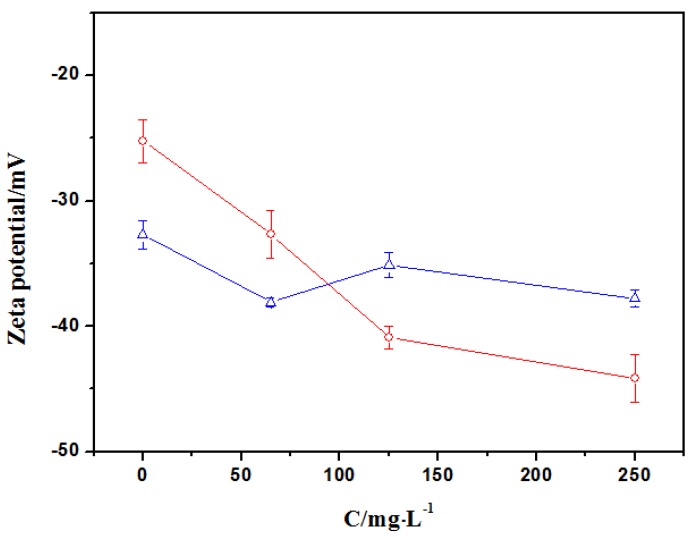
Effect of C_60_(OH)*_n_* and C_60_(C(COOH)_2_)*_n_* on the zeta potential of oMWCNTs. red line represent C_60_(OH)*_n_* effect; blue line represent C_60_(C(COOH)_2_)*_n_* effect.

The decreases of Th(IV) adsorption on oMWCNTs may be explained by a competition between C_60_(OH)*_n_* and Th(IV) for the sorption sites on the oMWCNTs surface. Both the adsorption of Th(IV) and C_60_(OH)*_n_* occurred on the surface of oMWCNTs, whereas Th(IV) cannot interact with C_60_(OH)*_n_*. In other words, C_60_(OH)*_n_*-oMWCNTs and Th-oMWCNTs complexes can be formed but C_60_(OH)*_n_*-Th may not formed. Figure 1B confirms that the adsorption of C_60_(OH)*_n_* takes place onto oMWCNTs. Figure A1 demonstrates that the zeta potential of oMWCNTs in solution decreases regularly with increasing concentration of C_60_(OH)*_n_*. This verified that the dispersivity of oMWCNTs in solution has been improved by adsorbed C_60_(OH)*_n_*. The adsorption of C_60_(OH)*_n_* on oMWCNTs depends on the strong π–π electron donor-acceptor interactions between the flat surfaces of both aromatic C_60_(OH)*_n_* and oMWCNTs [47]. The complexation between C_60_(OH)*_n_* and oMWCNTs is stronger than that between Th(IV) and oMWCNTs. Adsorbed C_60_(OH)*_n_* may block available sites of oMWCNTs surface which would be originally for the adsorption of Th(IV) [31]. With increasing pH, Th(IV) would be “squeezed” down by the increasing adsorption of C_60_(OH)*_n_* on oMWCNTs due to the enhancement of space hindrance.

From Figure 10, one can still see that at pH > 9, Th(IV) adsorption rises again slightly, which could be due to following factors: at pH > 9, Th(OH)_3_(CO_3_)^−^ and Th(CO_3_)_5_^6−^ is expected to begin forming and Th(OH)_4_ is the predominating species. Adsorbed C_60_(OH)*_n_* on oMWCNTs surface enhances electrostatic repulsion of the Th(OH)_3_(CO_3_)^−^ and Th(CO_3_)_5_^6−^ [31]. In general, the adsorption behavior of Th(IV) in the presence of C_60_(OH)*_n_* may be described as a competition between Th(IV) and C_60_(OH)*_n_* for the binding sites on the surface of oMWCNTs. Adsorbed C_60_(OH)*_n_* may also affect the adsorption of Th(IV) by altering surface charges and/or blocking available sites binding Th(IV). This assumption was not verified, and further work is in progress.

#### 2.3.2. Adsorption Isotherms at Constant Adsorbent Dose and Initial Concentration

**Figure 11:**
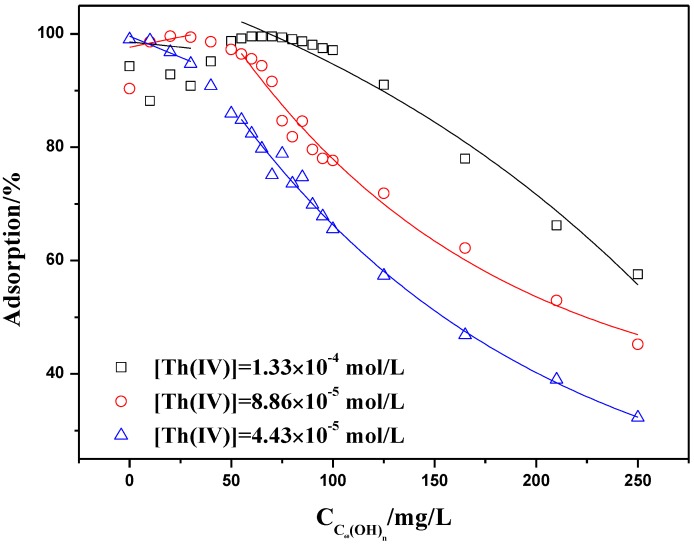
Effect of Th(IV) initial concentrations on Th(IV) adsorption onto oMWCNTs as a function of C_60_(OH)*_n_* initial concentrations, *m/V* = 0.1 g/L, pH = 3.20 ± 0.05, *I* = 0.01 mol/L NaNO_3_, *T =* 25 ± 1 °C.

**Figure 12:**
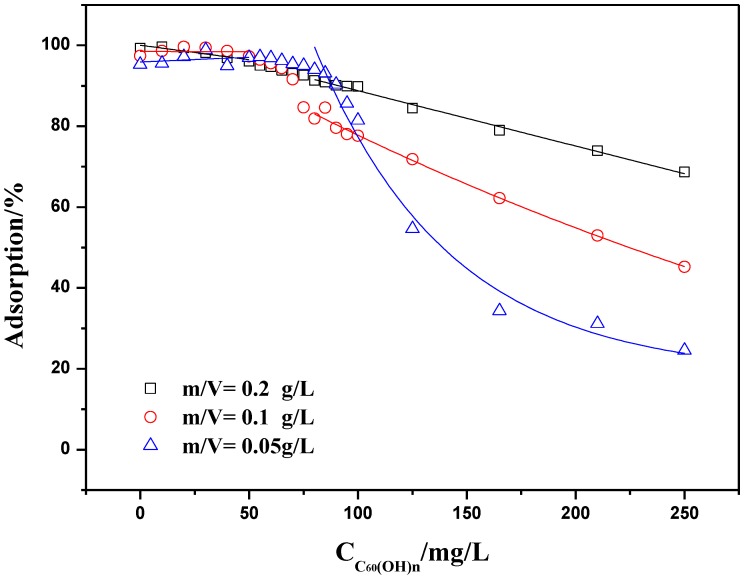
Effect of oMWCNT dosage on Th(IV) adsorption on oMWCNTs as a function of C_60_(OH)*_n_* initial concentrations, pH = 3.20 ± 0.05, *I* = 0.01 mol/L NaNO_3_, *T =* 25 ± 1 °C, C[Th^4+^] initial = 8.86 × 10^−5^ mg/L.

Adsorption experiments have been conducted at constant concentrations of Th(IV) and ratio of solid to liquid as a function of C_60_(OH)*_n_* concentration (as shown in Figure 11 and Figure 12). The C_60_(OH)*_n_* concentration varied from 0 to 250 mg/L. In a general manner, Th(IV) adsorption onto oMWCNTs decreases when C_60_(OH)*_n_* concentration increases whatever the concentrations of Th(IV) and ratio of solid to liquid is. The adsorption isotherms reveals a similar behavior as can be seen from Figure 10 and Figure 11 shows that Th(IV) adsorption decreases by half with the initial concentration of Th(IV) decreasing by half, indicating that Th(IV) does not interact with C_60_(OH)*_n_*. Figure 12 presents that when the dosage of oMWCNTs are 0.2 and 0.1 g/L and the concentration of C_60_(OH)*_n_* is less than 100 mg/L, the adsorption of Th(IV) on oMWCNTs maintains stable at a high level, then falls sharply when the concentration of C_60_(OH)*_n_* exceeds 100 mg/L. As the dosage of oMWCNTs is 0.05 g/L, Th(IV) adsorption decreases gradually when the concentration of C_60_(OH)*_n_* is more than 100 mg/L and remains at a minimum value of ~25%. This may be demonstrates indirectly the adsorption of C_60_(OH)*_n_* on oMWCNTs.

Both of the experimental curves in Figure 11 and Figure 12 can be divided into two distinct regions. In the first region, where the concentration of C_60_(OH)*_n_* is lower than 50 mg/L, Th(IV) adsorption is more than 90%; In the second region, where the concentration of C_60_(OH)*_n_* is higher than 50 mg/L, the adsorption of Th(IV) onto oMWCNTs is decreased with increasing C_60_(OH)*_n_* concentration. That is to say, C_60_(OH)*_n_* suppresses the Th(IV) adsorption when its concentration exceeds a critical value.

### 2.4. Influence of C_60_(C(COOH)_2_)_n_ on Th(IV) Adsorption

**Figure 13:**
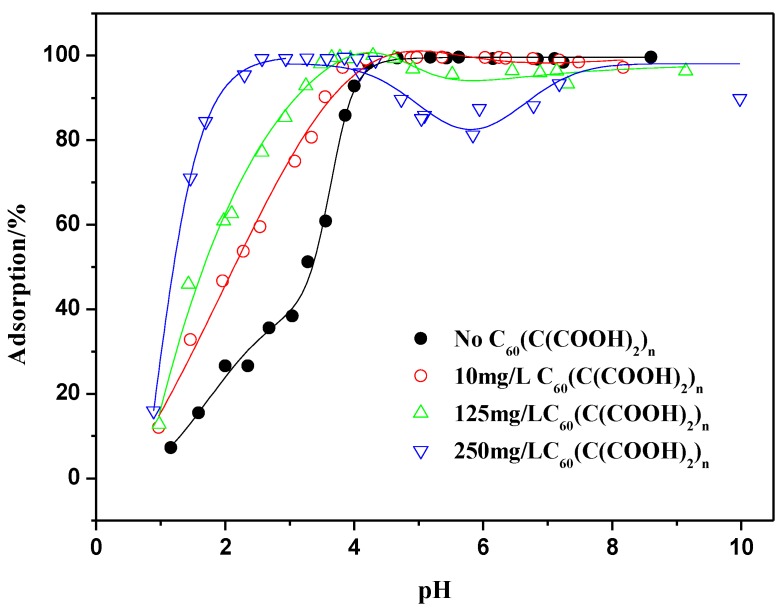
Effect of C_60_(C(COOH)_2_)*_n_* on Th(IV) adsorption onto oMWCNTs as a function of pH, *m/V* = 0.1 g/L, *T =* 25 ± 1 °C, *I* = 0.01 mol/L NaNO_3_, C[Th^4+^] initial = 8.86 × 10^−5^ mol/L.

Figure 13 exhibits the adsorption of Th(IV) on oMWCNTs as a function pH in the presence and absence of C_60_(C(COOH)_2_)*_n_*. The adsorption curve of Th(IV) onto oMWCNTs in the presence of C_60_(C(COOH)_2_)*_n_* shifts to the left as compared to that in the absence of C_60_(C(COOH)_2_)*_n_* at pH < 4, from which it can be concluded that the presence of C_60_(C(COOH)_2_)*_n_* enhances Th(IV) adsorption at pH < 4. Moreover, the adsorption of Th(IV) increases with increasing of C_60_(C(COOH)_2_)*_n_* concentration. It can conjectured that the complexation between C_60_(C(COOH)_2_)*_n_* and Th(IV) is formed. The complexes may promote the precipitation of Th(IV) on the surface of oMWCNTs [37]. Therefore Th(IV) adsorption on oMWCNTs is enhanced in the presence of C_60_(C(COOH)_2_)*_n_* in ternary system. But with increasing of pH, one can see that Th(IV) adsorption reduces slightly at intermediate pH (~4–6) and then rises again until equilibrium at pH > 6. This also indicates that the negative effect of C_60_(C(COOH)_2_)*_n_* on Th(IV) sorption is different from that of C_60_(OH)*_n_*. The steric hindered effect of C_60_(C(COOH)_2_)*_n_* is stronger than that of C_60_(OH)*_n_*, and C_60_(C(COOH)_2_)*_n_* can not easily be adsorbed onto oMWCNTs like C_60_(OH)*_n_*. This may be the reason why no C_60_(C(COOH)_2_)*_n_* connected to oMWCNTs surface is observed in the TEM photos of the dispersion of oMWCNTs and C_60_(C(COOH)_2_)*_n_* (seen from Figure 1C). However, C_60_(C(COOH)_2_)*_n_* can adsorbed on oMWCNTs surface due to the interaction of their functional groups at intermediate pH, which results in the increasing space hindrance of C_60_(C(COOH)_2_)*_n_*, thus reducing the Th(IV)adsorption.

## 3. Experimental Section

### 3.1. Materials

MWCNTs (prepared by chemical vaporization deposition) were purchased from Shenzhen Nanotech Port Co., Ltd. China. According to the product specification, the as-grown MWCNTs are 1−10 μm in length, with outer diameter range of 10−30 nm, and purity above 96% (including amorphous carbon < 3%, ash < 0.2%). Oxidized MWCNTs were prepared by oxidization with a mixture of concentrated nitric acid and sulfuric acid (1:3, V/V) [48]. The surface area of oMWCNTs is 100 m^2^/g determined using N_2_-BET method (micromeritics Surface Area and Porosity Analyzer, ASAP 2020, American). Chen *et al.* [49] have measured the surface area of raw carbon nanotubes and the result was 86 m^2^/g. This indicates that the oxidation process improves the specific surface area of the MWCNTs.

Fullerenes (C_60_), purity > 99%, was bought from Henan Puyang Yongxin Fullerene Technology Co., LTD. C_60_(OH)*_n_* (*n* = 2~24) was synthesized in the procedure reported by Li *et al.* [50]. Synthesis of C_60_(C(COOH)_2_)*_n_* has been described in the literature [51].

All chemicals were of analytical grade and all solutions were prepared using deionized water.

### 3.2. Adsorption Experiments

The adsorption of Th(IV) on oMWCNTs was investigated using batch technique in 10 mL polyethylene centrifuge tube. The stock solutions of oMWCNTs and NaNO_3_ were mixed and pre-equilibrated for 24 h and then the Th(IV) stock solution was added to achieve the desired concentrations of different components. The system was adjusted to the desired pH by adding negligible volumes of 0.01 or 0.1 mol·L^−1^ HNO_3_ or NaOH. After the suspensions were gently shaken for 24 h (which was enough to achieve equilibrium), the solid and liquid phases were separated by using centrifugating at 12,000 rpm for 30 min. The concentration of Th(IV) in the aqueous solution was analyzed using the thorium arsenazo(III) complex method with UV-VIS spectrophotometer (Perkin-Elmer, American) at 654 nm. The amount of Th(IV) adsorption was calculated from the difference between the initial concentration and the equilibrium one.

Adsorption isotherms were investigated by using batch technique in polyethylene centrifuge tubes under ambient conditions at 293, 313, and 333 K, respectively. The stock solutions of 0.01 mol/L NaNO_3_ and 0.5 g/L oMWCNTs were mixed and pre-equilibrated for 24 h before the addition of Th(IV) stock solution. The initial concentrations of Th(IV) varied from 8.86 × 10^−6^ to 8.86 × 10^−4^ mol/L.

The effect of C_60_(OH)*_n_*/C_60_(C(COOH)_2_)*_n_* on the adsorption of Th(IV) was also investigated. The stock solutions of oMWCNTs and C_60_(OH)*_n_*/C_60_(C(COOH)_2_)*_n_* and NaNO_3_ were mixed and pre-equilibrated for 24 h before adding the Th(IV) stock solution. Other procedures were the same as the single-solute sorption experiments.

## 4. Conclusions

The results of the present work indicated that chemical complexation was the main mechanism of Th(IV) adsorption onto oMWCNTs. The competition adsorption between C_60_(OH)*_n_* and Th(IV) may be attributed to the negative effects of C_60_(OH)*_n_* on Th(IV) adsorption onto oMWCNTs. While C_60_(C(COOH)_2_)*_n_* exhibited positive effect may be due to the fact that C_60_(C(COOH)_2_)*_n_* promoted the precipitation of Th(IV). The results are important to understand the fate and transport of Th(IV) from two kinds of carbon nano-materials and to evaluate their behavior in the environment. However, new detection method need to be developed for testing the adsorption of C_60_(OH)*_n_* and C_60_(C(COOH)_2_)*_n_* on oMWCNTs. Further systematic investigation is needed to understand fully the sorption mechanism. Such studies are continuing and will be the subject of future articles.
